# Don’t put words in my mouth: speech perception can falsely activate a brain-computer interface

**DOI:** 10.1186/s12984-025-01689-7

**Published:** 2025-08-19

**Authors:** Anouck Schippers, Mariska J. Vansteensel, Zac V. Freudenburg, Shiyu Luo, Nathan E. Crone, Nick F. Ramsey

**Affiliations:** 1 Department of Neurology and Neurosurgery, University Medical Center Utrecht Brain Center, Heidelberglaan 100, Utrecht, 3584 CX The Netherlands; 2 Department of Biomedical Engineering, Johns Hopkins University School of Medicine, Baltimore, MD USA; 3 Department of Neurology, Johns Hopkins University School of Medicine, Baltimore, MD USA; 4 Donders Institute for Brain, Cognition and Behavior, Radboud University, Nijmegen, The Netherlands

**Keywords:** Speech decoding, Brain-Computer interface, Neuroprosthesis, Reliability, Communication, Locked-in syndrome, ALS

## Abstract

**Background:**

Recent studies have demonstrated that speech can be decoded from brain activity which in turn can be used for brain-computer interface (BCI)-based communication. It is however also known that the area often used as a signal source for speech decoding BCIs, the sensorimotor cortex (SMC), is also engaged when people perceive speech, thus making speech perception a potential source of false positive activation of the BCI. The current study investigated if and how speech perception may interfere with reliable speech BCI control.

**Methods:**

We recorded high-density electrocorticography (HD-ECoG) data from five subjects while they performed a speech perception and a speech production task. We first evaluated whether speech perception and production activated the SMC. Second, we trained a support-vector machine (SVM) on the speech production data (including rest). To test the occurrence of false positives, this decoder was then tested on speech perception data where every perception segment that was classified as a produced syllable rather than rest was considered a false positive. Finally, we investigated whether perceived speech could be distinguished from produced speech and rest.

**Results:**

Our results show that both the perception and production of speech activate the SMC. In addition, we found that decoders that are highly reliable at detecting self-produced syllables from brain signals may generate false positive BCI activations during the perception of speech and that it is possible to distinguish perceived speech from produced speech and rest, with high accuracy.

**Conclusions:**

We conclude that speech perception can interfere with reliable BCI control, and that efforts to limit the occurrence of false positives during daily-life BCI use should be implemented in BCI design to increase the likelihood of successful adoptation by end users.

**Supplementary Information:**

The online version contains supplementary material available at 10.1186/s12984-025-01689-7.

## Background

Neurodegenerative conditions such as amyotrophic lateral sclerosis (ALS) can lead to severe paralysis, to the point where individuals enter a locked-in state where they are completely unable to voluntarily move and speak but remain cognitively intact [1, 2]. For these individuals, a Brain-Computer Interface (BCI) may provide a new means of communication. A BCI allows a user to control a computer by modulating their brain activity, for example by attempting to move their hand or produce speech. In this way, an individual with severe paralysis can operate a device and spell letters, words, or even produce full sentences.

Recent developments in the field of implanted BCIs have demonstrated their huge potential as a communication solution for people with severe motor impairment. It has been demonstrated that BCIs can be used by individuals with ALS to control a communication application on a computer by means of a binary click based on attempted hand movements [3, 4] with long-term stability [5]. Another way to control a device is through discrete speech commands to navigate on a computer screen [6]. Others have focused on direct speech decoding for communication. Using subdural electrocorticography (ECoG) grids to record cortical activity during attempted speech production in a severely paralyzed person with minimal preserved articulation, words and full sentences were successfully decoded with a limited vocabulary [7] as well as sentences in a large vocabulary by spelling individual letters [8]. ECoG-based activity patterns during speech production can also directly be synthesized into speech sounds [9–11], or combined with decoding of discrete words into a multimodal speech prosthesis [12]. Intracortical approaches with indwelling microelectrode arrays have also been used with success to control a speech BCI, decoding speech similar to the ECoG studies [13, 14].

Incorporating the needs and wishes of potential end-users is of utmost importance for clinical and societal adoption of BCIs. Efficiency and user satisfaction are two factors determining usability, but especially in situations where a BCI is used for communication and device control, high reliability of the device is crucial [15]. Accuracy and communication speed are the key metrics in BCI, such as in the studies described above, but attention for reliability in real-life settings has been underwhelming. A reliable BCI should decode each communication-attempt that is intended (true positives) and remain silent when the user is not intending to communicate (true negatives). A BCI that is unreliable would miss communication attempts (false negatives) and produces BCI activations or utterances that the user was not intending to produce (false positives). In the case of speech BCIs, false positives may occur when brain activity patterns outside of attempts to speak resemble those decoded during speech production. If a BCI is too sensitive and also labels brain activity other than attempted speech as communication attempts, the resulting false positives will disrupt conversation or cause confusion about the needs and wishes of the user. A high false positive rate may even cause caregivers to disregard BCI output. Designing an accurate system that retains a low false positive rate in all circumstances is thus of utmost importance for speech BCIs to be successfully adopted by envisioned end users.

Most speech BCIs are based on brain signals originating from the sensorimotor cortex (SMC, pre- and postcentral gyri), of which the most ventral part is activated during the movement of the articulators [16]. However, this area also exhibits activation during perception of speech [17–21] which may well pose a source of false positive activation of a speech BCI. A recent article reported overall high reliability of a speech BCI decoder, also in conversational settings, but of all trials in which speech was detected, in a small percentage the participant was not attempting to speak [22]. To the best of our knowledge, no published information is available on a structured investigation of the performance of speech decoders during the perception of speech.

Considering that speech perception is a vital part of human interaction and communication, it is important to verify if and how it may interfere with speech BCI control. The current study was conducted to investigate similarities between brain activity patterns during speech perception and production, and to assess whether decoders designed to detect and classify produced speech generate false positives during the perception of speech. Our first objective was to identify which areas of the SMC are responsive to speech perception and/or speech production. In our second objective, we evaluated to what extent perceived speech was classified as produced speech (i.e., false positive activations), using a decoder trained solely on produced speech. Finally, the third objective was to investigate whether a classifier was able to distinguish between speech perception, speech production, and rest, which could pave the way for strategies to limit the occurrence of false positives in daily-life speech BCI use. Given that end-users of BCI implants for communication will not be able to generate sounds, we conducted the analyses for overt as well as for mimed (silent) produced speech.

## Methods

### Participants

Five participants (mean age 33.4 years, two females, all right-handed, see Table 1) who underwent epilepsy treatment at the University Medical Center Utrecht were included in the current study. As part of their presurgical assessment, they were implanted with subdural clinical ECoG electrode grids. They gave written informed consent to participate in this study and for the implantation of an additional HD electrode grid, which was placed over the SMC. Only the ECoG data acquired with these HD grids are used in this study. The study was conducted in accordance with the Declaration of Helsinki (2013) and approved by the Medical Ethical Committee of the University Medical Center Utrecht.

**Table 1:** Participant demographics and timeline^a^ of data acquisition

Participant	Age at implant	Sex	Speech perception recording	Speech production recording	Rest recording
1	24	M	Day 2	Day 2	Day 3
2	40	F	Day 4	Day 7	Day 5
3	31	M	Day 1	Day 1	Day 2
4	46	F	Day 2	Day 3	Day 3
5	26	M	Day 1	Day 2	Day 2

^a^Days after electrode grid implantation

### Task design

All participants performed a speech perception and a production task, which both included the same sequence of syllables: “Do”, “Re”, “Mi”, “Fa”, “So”, “La”, and “Ti” (see Supplementary Figure 1). This sequence of syllables was chosen since it was expected to be well known to the participants, thus removing the need for training and risk of errors and limiting the cognitive processes occurring during the tasks. Each trial comprised the full sequence of seven syllables. Both speech tasks were about 9 min in duration. In both tasks, active trials were alternated with rest trials, indicated by a fixation cross which was presented for 2.5 s. A 3-minute rest task was recorded in addition to the speech tasks, where participants were instructed to focus on a fixation cross while refraining from generating movements. The tasks were presented using the Presentation^®^ Software (Neurobehavioral systems, Inc., Berkeley, CA), on a laptop placed about 1 m in front of the participant.

In the perception task, the speech stimuli were presented in an audiovisual, visual-only, and audio-only fashion. Only the audiovisual data were used for the current report since these best simulate a real-life communication setting. In ten audiovisual trials the lower half of a woman’s face was presented on the screen while she monotonally produced the syllables, with about 1.75 s intervals. Participants were instructed to attentively watch the screen and listen to the syllables, but not produce any movements or sounds themselves. Video and audio were acquired during task execution to verify that no mouth movements or sounds were made during the task. The rest trials corresponding to this task will further be referred to as the perception-rest trials.

In the speech production task, participants were instructed to produce the same sequence of syllables, with each complete sequence constituting one trial. Prior to each trial, an instruction on the screen (1.5 s duration) indicated if participants should produce the sequence in an overt, whispered, or mimed manner. Each condition was presented ten times, in random order. Participants were cued to produce the sequence of syllables with 1.75 s intervals, using a rotating cursor. The first syllable (“Do”) was cued 1.75 s after the instruction. Participants were instructed to produce the syllables monotonally, and audio and video recordings were inspected to confirm monotonal pronunciation. In this study, only the overt and mimed production trials were used, in addition to rest, since these manners of production are commonly used in speech decoding, and since end-users of BCI implants for communication will not be able to generate sounds. The rest trials corresponding to this task will further be referred to as the production-rest trials.

### Electrode locations

**Fig. 1:**
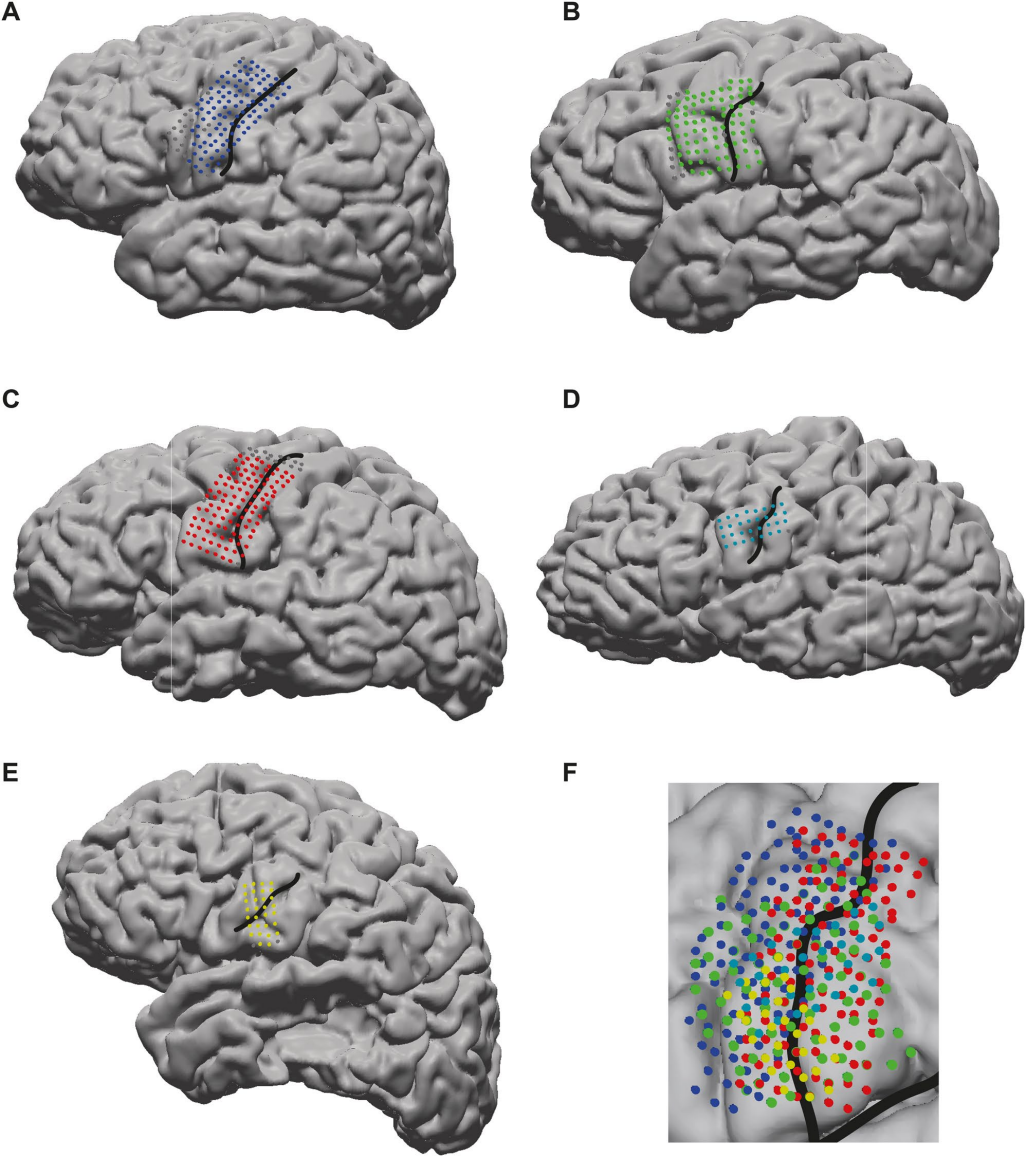
Grid placement. (**A-E**) Grid placement on individual surface reconstructions for participants 1–5. Central sulcus is indicated with a black line. Grey electrodes are excluded because they showed poor signal quality or were outside of the SMC. (**F**) Electrode placement (coordinates converted to MNI space) of all included electrodes of all participants, projected to an MNI cortex reconstruction. Central sulcus and Sylvian fissure are indicated with black lines

The HD ECoG grids were placed over the face area of the left SMC, which was adjacent to the area of clinical interest. A pre-implantation MRI and a post-implantation CT scan were used to determine the exact location of the grids using the ALICE toolbox [23]. Using Freesurfer surface reconstructions of each individual brain and the electrode coordinates, each electrode was assigned to an anatomical region according to the Allen atlas (see Fig. 1 for electrode placements and Table 2 for information on the implanted grids). Electrode coordinates were converted to Montreal Neurological Institute (MNI) space to allow for group-analysis in standard space.

**Table 2:** Grid and electrode information

Participant	Grid manufacturer	Electrode diameter	Inter-electrode distance	Total electrodes	Electrodes with bad signal quality	Final included electrodes
1	PMT Corporation	1 mm	3 mm	128	9	105
2	AD-Tech Medical Instrument Corporation	1.17 mm	3 mm	96	3	84
3	PMT Corporation	1 mm	3 mm	128	18	108
4	CorTec	1 mm	4 mm	32	0	31
5	CorTec	1 mm	4 mm	32	1	30

### Data acquisition

For participants 1–3, a 256-channel Blackrock Neuroport system (Salt Lake City, USA) was used to record the HD-ECoG signals at a sampling frequency of 2000 Hz (band pass filtered between 0.3 and 500 Hz). Audio was recorded simultaneously on the Neuroport system, at a sampling frequency of 30 kHz. For participants 4 and 5, the HD-ECoG was recorded on a Micromed system (Treviso, Italy) at a sampling frequency of 2048 Hz (band pass filtered between 0.15 and 926.7 Hz), and audio was recorded in the task presentation software (Presentation Neurobehavioral Systems) at 44.1 kHz. For all participants, the brain recordings were referenced to a clinical reference and a ground electrode placed on the forehead and mastoid. Video recordings were made on the Micromed system during task execution and video, audio, and HD-ECoG recordings were aligned using a beep presented at the start of each task and an event marker that was sent to the recording system.

### Data processing

The HD-ECoG data was tested for acoustic contamination following the methods provided by Roussel et al. [24] No signs of acoustic contamination were found in the speech perception and production data for all participants.

The data was preprocessed by first applying a notch filter to remove line noise at 50 and 100 Hz. Second, electrodes that were located on top of a clinical grid, facing the dura, or those showing poor signal quality (e.g., excessive noise, flat signals) were identified and excluded from further analysis (see Table 2 for number of electrodes per participant). Third, a common average re-reference (CAR) was applied on the remaining electrode signals. The resulting preprocessed data was downsampled to 500 Hz.

To extract the spectral response for each electrode, a Gabor Wavelet Dictionary was used. The ECoG signal was convolved with Gabor wavelets with a full-width half-maximum of four wavelengths in each individual frequency from 1 to 130 Hz. To compute the power response within the high-frequency band (HFB) the log of the sum of the absolute values was taken within the frequency range of 65 to 95 Hz. The resulting power responses were normalized by z-scoring the signals using the mean and standard deviation of signals recorded during the three-minute rest task. Finally, the power responses were smoothed over a window of 50 ms. Only electrodes positioned over the SMC (pre- and postcentral gyrus) were included in further analysis.

### Speech epoch extraction

For each individual syllable, the power trace within a period of interest around speech onset was extracted from the HFB power signals, which will be referred to as a speech epoch. For the perception data, stimulus onset was defined as the moment the syllable could be audibly perceived. The period of interest for the perception task is 0 to 700 ms after stimulus onset. For the production task, speech onset was defined as the first moment the syllable could audibly be perceived. For the mimed syllables, the speech onset was determined as the first moment a speech movement could be observed on the video recordings. The period of interest for the production task was 200 ms before until 500 ms after speech onset.

A maximum of 70 active speech epochs were extracted from the power data for each task. Production epochs in which participants did not produce the correct syllable were excluded (six and seven overt epochs for participants 2 and 4, respectively, five, eleven and one mimed epochs for participants 1, 2, and 4, respectively). No perception epochs had to be excluded.

In the production and perception tasks, seven rest epochs of 700 ms were extracted from each 2.5 s rest trial by randomly sampling seven time periods that fell within the rest trial. Doing this, a maximum of 210 rest epochs could be extracted per task (less if there was noise or movement during rest epochs: seven perception-rest epochs were excluded for participants 1, 4, and 5; seven production-rest epochs for participants 4 and 5, and 42 production-rest epochs for participant 2).

### Objective 1: SMC engagement during speech perception and production

To confirm correct placement of the grids for the tasks, the correlation between HFB power in trials and the task conditions (ones for active trials, zeros for rest trials) was assessed by means of coefficient of determination (R^2^). For active trials, the mean power over all seven epochs of 700 ms within that trial was used, for the rest trials the mean over the entire 2.5 s rest period. The significance of the R^2^ values was determined at *p* <.05, Bonferroni corrected for the number of included SMC electrodes per participant.

### Objective 2: Classification of produced and perceived speech epochs

The above-described procedure of speech epoch extraction resulted in an imbalanced number of epochs between syllables and rest. The imbalance was maintained for the classification procedure to resemble the daily-life use situation, where there are more moments during which a BCI user is not intending to communicate speech versus when they are.

For classification of the limited number of discrete classes of the speech tasks, a support vector machine (SVM) decoder was trained separately for overt and the mimed speech [25–28]. For this purpose, the mean power response over time per electrode was calculated for each production and production-rest epoch. Then, a Leave-one-Out (LoO) approach was used to decode each epoch (eight-way classification: seven syllables and rest). Specifically, an SVM was trained on all but one epoch, and then tested on the left-out epoch. After doing this for all individual production (active and rest) epochs, decoding accuracy could be calculated by dividing the number of correctly classified epochs by the total number of epochs and multiplying that by 100 to get a percentage score. Since numbers of epochs were not equal between classes due to discarded epochs and ratio of active versus rest epochs, a permutation test was performed for each participant for the production task, to obtain estimates of actual chance levels and significance of classification. The production labels were randomly shuffled, and production epochs were again classified using those shuffled labels with a LoO approach. This was repeated a total of 1000 times, which generated a distribution of random classification scores. The classification accuracy using the unshuffled labels was compared to the random distribution and considered significantly above chance if it fell in the upper 5% (*p* <.05) of random classification scores.

In a real-word situation, a decoder based on produced speech should not activate during the perception of speech. To test whether a decoder based on produced speech would generate false positives during perception, an SVM was trained on all production (active and rest) epochs, again separately for the overt and the mimed produced epochs. All perception and perception-rest epochs were then classified as either one of the syllables or as rest based on this decoder. All perception and perception-rest epochs that were classified as anything else than a rest epoch were considered false positives, since the participants were not producing any speech movements or sounds throughout the task. The percentage of false positives was calculated by dividing the number of non-rest classifications by the total number of epochs and multiplying this by 100.

To verify whether the false positive epochs were caused by baseline differences in the task recordings (caused by for example day-to-day variations in power amplitudes), we repeated the analysis after normalizing all epochs based on the rest data within each task. For this, the mean and standard deviation was calculated per electrode over all 2.5 s rest periods within each task. Then, each epoch was z-scored by subtracting the mean from each timepoint and dividing this by the standard deviation. This rest normalization brought the power signals from different tasks within the same range. The same steps as described above were then taken to determine the classification accuracy on production epochs (and significance of this score) and the percentage of false positives during the perception epochs, now using the z-scored epochs.

### Objective 3: Distinguishing speech perception from production and rest

Identification of a brain activity pattern as produced or perceived speech might help mitigate the problem of false positive activations due to speech perception. Therefore, to investigate the differences between brain activity patterns during speech perception and production, we tested if the two brain states could be distinguished from each other and from rest. This was using the rest-normalized epochs from both the perception and production task, since after rest normalization the power amplitudes from the different task recordings were brought to the same range, allowing the rest epochs from the two different tasks to be grouped. Using a LoO approach, each epoch was classified as being a speech perception, production, or rest epoch, based on an SVM trained on all other epochs. Classification accuracy was determined by dividing the number of correctly classified epochs by the total number of epochs and multiplying this by 100 to get a percentage score. Significance of classification score was obtained with permutation testing, similar to how this was done for objective 2 as described above, and a threshold of 0.05.

## Results

### Objective 1: SMC engagement during speech perception and production

**Fig. 2:**
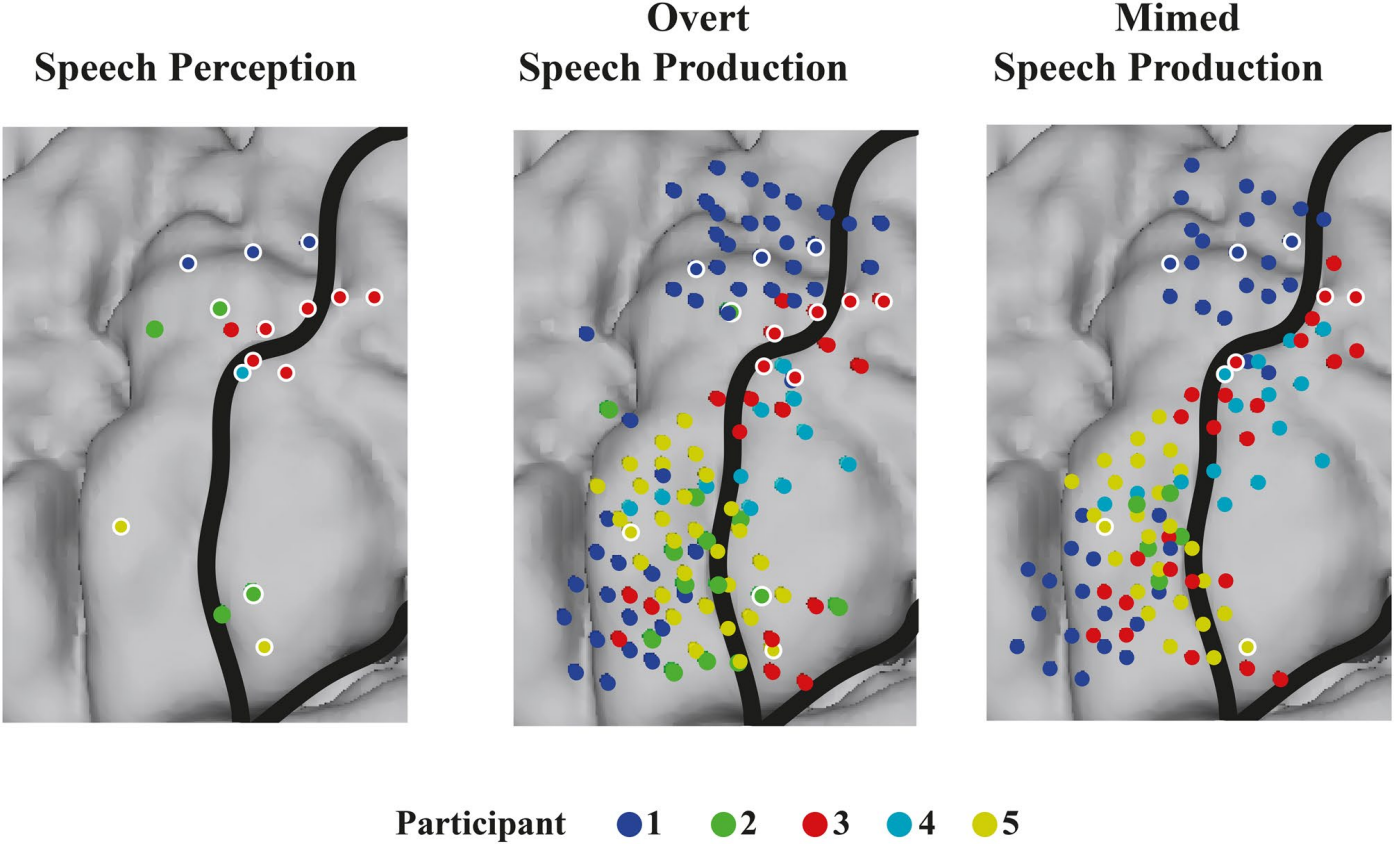
Significantly responding electrodes to the tasks for all subjects. Electrodes are visualized on an MNI brain. Central sulcus and sylvian fissure are indicated with black lines. Different participants are indicated in different colors. Speech perception electrodes that are responsive during at least one version of production are indicated with a white rim (left panel), as are production electrodes that are also responsive during speech perception (middle and right panel)

All participants showed engagement of the SMC during both speech perception and production, as quantified by having at least one electrode with a significant increase in HFB power during the active periods (syllables) compared to rest. Locations of activity partially overlapped between tasks (see Fig. 2). Of the electrodes that were active during overt production, on average 10.87% were also active during speech perception (3/49, 2/14, 6/22, 0/11, and 2/30 electrodes for participants 1–5, respectively). For mimed production, on average 6.7% of electrodes were also active during perception (3/42, 0/5, 3/25, 1/15, and 2/26 for participants 1–5, respectively).

### Objective 2: Classification of produced and perceived speech epochs

#### Classification of overt syllables

**Fig. 3:**
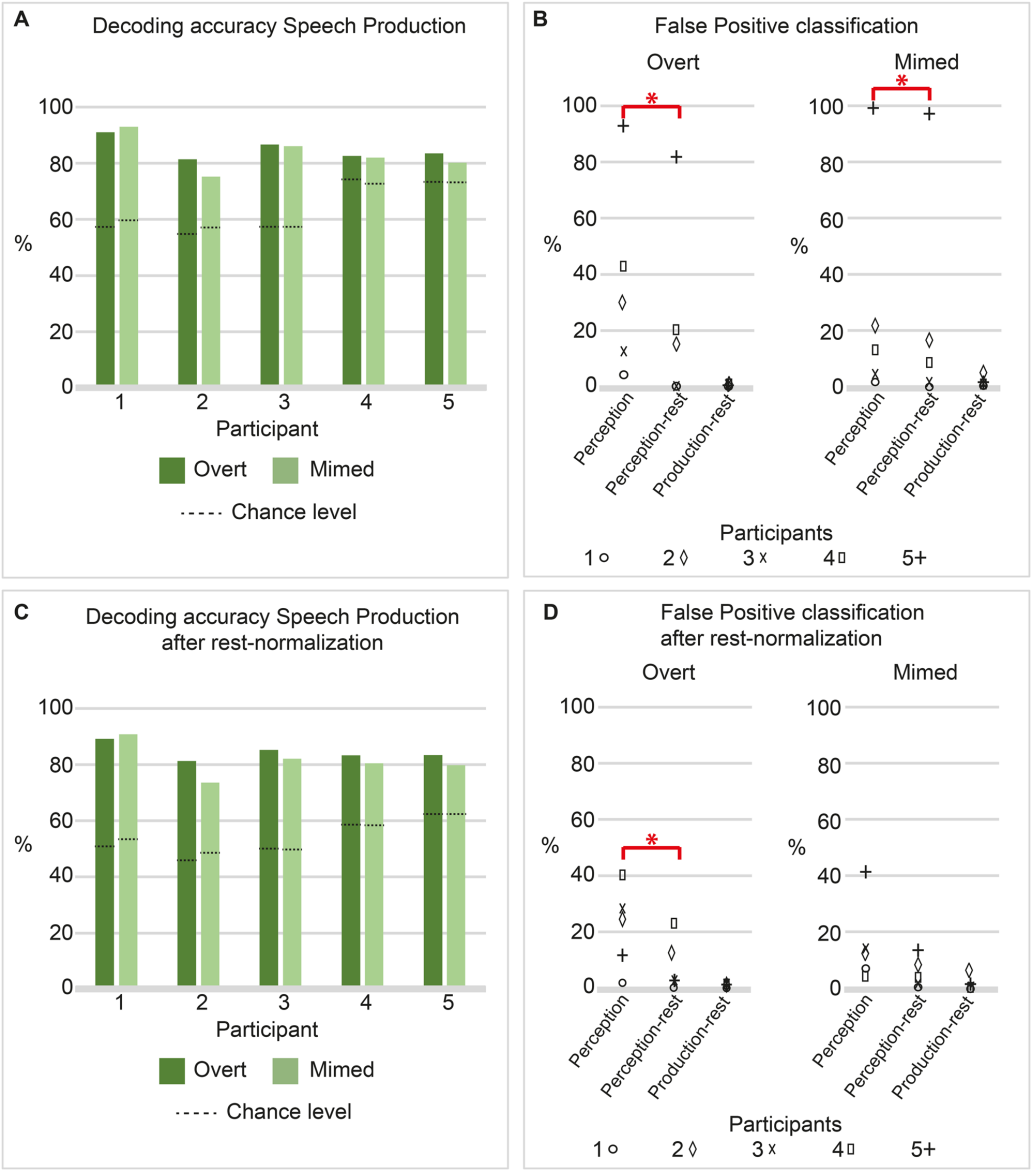
Classification scores and False Positive rates. (**A**, **C**) Classification scores on produced speech epochs for each participant using an overt-based (dark green) and mimed-based (light green) decoder, before (**A**) and after (**C**) rest normalization. Dashed lines indicate the chance level, which is the mean classification accuracy of the distribution generated using random labels. (**B**, **D**) Percentages of false positively classified epochs for the decoder based on the overt and mimed speech separately, before (**B**) and after (**D**) rest normalization. The percentage scores of false positive classified epochs are visualized for the perception epochs (left), the perception-rest epochs (middle), and the production-rest epochs (right). Subjects are indicated with different markers. Red bars indicate significant differences, with * indicating *p* <.05

The overt produced syllables were classified above chance for all participants (*p* <.01, mean chance level 63.42%), with an average decoding accuracy of 85.11% (ranging between 81.47 and 91.07%, Fig. 3A, Supplementary Figure S2, Supplementary Figure S4). Considering the imbalance in active and rest epochs, the chance level of overt syllable decoding was well above the theoretical chance level of 12.5% (100 divided by eight classes). On average 0.72% of the production-rest epochs were classified as an overtly produced syllable (production false positive, Fig. 3B). Normalizing the epochs based on the mean and standard deviation of the rest periods during the production task resulted in slightly different (but significant, *p* <.01, mean chance level 53.59%) decoding accuracies: 84.24% (range between 81.03 and 88.93%, an average decrease of 0.87% points, Fig. 3C). An average of 0.82% of production-rest epochs were classified as an overtly produced syllable after normalization (Fig. 3D).

#### False positive classification during speech perception using a decoder based on overt speech

To assess sensitivity of the overt decoders to perceived speech, two types of additional epochs were tested, being perception and perception-rest. Applying the overt-based classifier on perception data generated false positives in all participants. On average, 36.57% (range 4.29 − 92.86%) of speech perception epochs were classified as produced syllables, while an average of 23.34% (range 0.00 – 81.77%) of perception-rest epochs were classified as such (Fig. 3B). All epochs that were classified as a produced syllable were considered false positives, regardless of the syllable class it was assigned to. A one-sided Wilcoxon signed rank test confirmed there were significantly more false positives during perception epochs compared to perception-rest epochs (*p* <.05), but not significantly more during perception-rest compared to production-rest (*p* = .13). After rest normalization, speech perception epochs were considered false positives in on average 21.14% of epochs (range 1.43 − 40.00%), which was again significantly higher *(p* <.05) than the false positives during perception rest epochs: 7.50% (range 0.00 − 22.17%, Fig. 3D). There were not significantly more false positives during perception-rest compared to production-rest (*p* = .06). Comparing the percentage scores of misclassified perception epochs before and after rest normalization, there is no consistent pattern over subjects. In four participants (1, 2, 4, and 5) there were fewer false positives after rest normalization, while in participant 3 the number of misclassified perception epochs increased. For the perception-rest epochs, there was again no consistent pattern of change after rest normalization. The percentage scores of false positive perception-rest epochs remained similar before and after normalization in participant 1, decreased in participants 2 and 5, but increased in participants 3 and 4.

#### Classification of mimed syllables

The mimed syllables were classified with an average classification accuracy of 83.34% (range 75.33 – 93.09%, Fig. 3A, Supplementary Figure S3, Supplementary Figure S5), which was significantly above chance for all participants (*p* <.01, mean chance level 64.00%). Considering the imbalance in active and rest epochs, the chance level of overt syllable decoding was well above the theoretical chance level of 12.5% (100 divided by eight classes). On average 1.44% of the production-rest epochs were classified as a produced syllable, and therefore considered a production false positive (Fig. 3B). After normalization, classification accuracy of the mimed syllables was on average 81.78% (range 74.01 – 91.27%, average decrease of 1.41% points, Fig. 3C), which was again above chance (*p* <.01, mean chance level 54.52%). On average, 1.89% of production-rest epochs were considered a production false positive (Fig. 3D).

#### False positive classification during speech perception using a decoder based on mimed speech

To assess sensitivity of the mimed decoders to perceived speech, two types of additional epochs were tested, being perception and perception-rest. All epochs that were classified as a produced syllable were considered false positives, regardless of the syllable class it was assigned to. For mimed speech, an average of 27.71% (range 1.43 − 98.57%) of speech perception epochs were misclassified as a produced syllable, versus 24.51% (range 0.00 − 96.55%) of the perception-rest epochs (Fig. 3B). A one-sided Wilcoxon signed rank test found significantly more false positives during perception compared to perception-rest epochs (*p* <.05), but not significantly more during perception-rest compared to production-rest (*p* = .06). After rest normalization, the average percentages false positive classified epochs were 16.00% (range 4.29 − 41.43%) and 5.84% (0.49 − 13.79%) for perception and perception-rest epochs, respectively (Fig. 3D). Here, there was no significant difference in false positives between perception compared to perception-rest epochs (*p* = .06), but there were significantly more during perception-rest compared to production-rest (*p* <.05). Comparing the percentage scores of misclassified perception epochs before and after rest normalization, rest normalization decreased the percentage score of misclassified perception epochs in three participants (2, 4 and 5), while these percentage scores increased in the other two (1 and 3). For the perception-rest epochs, the scores decreased in three (2, 4, and 5), and increased in participants 1 and 3.

### Objective 3: Distinguishing speech perception from production and rest

**Fig. 4:**
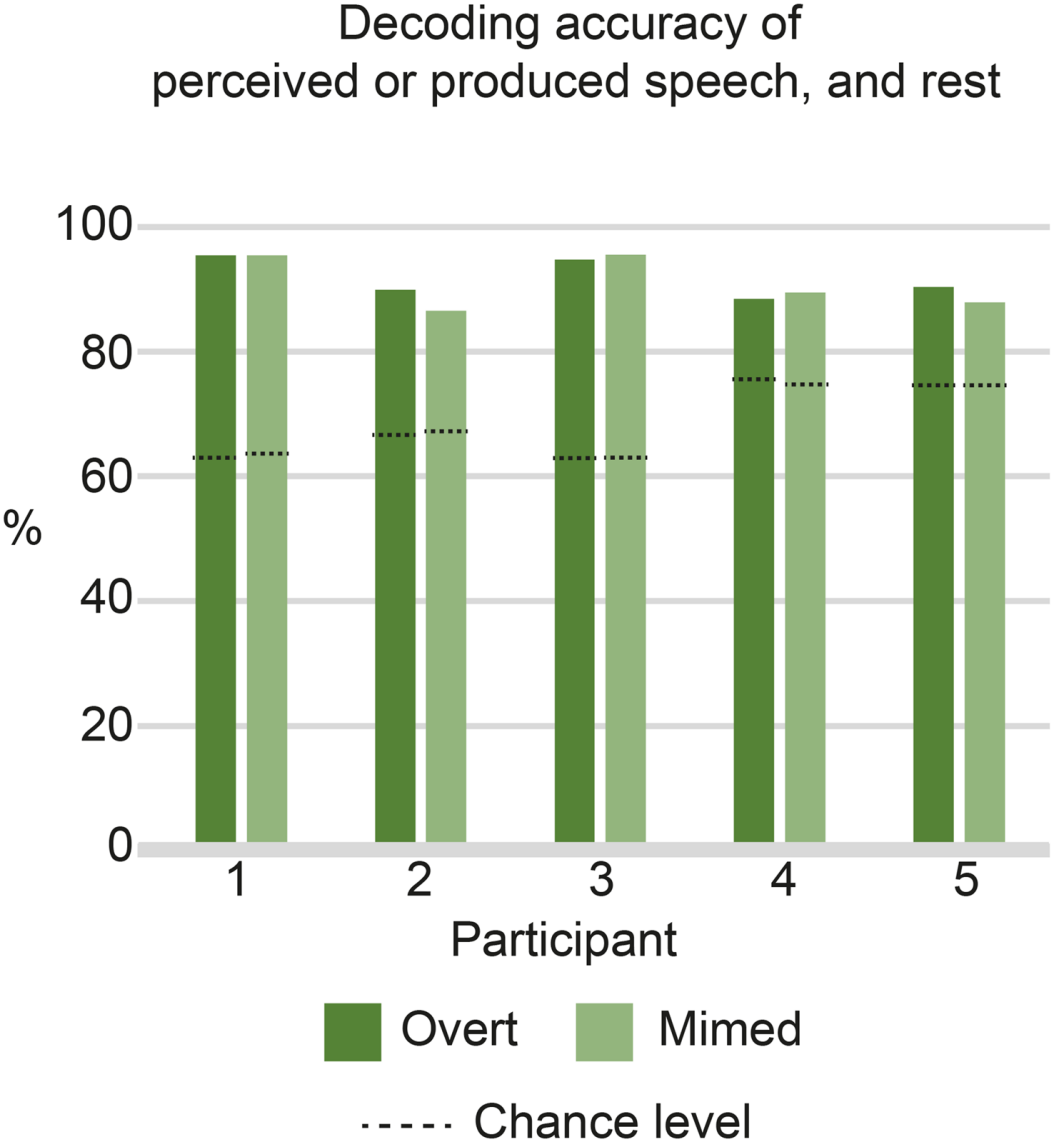
Decoding performance in distinguishing perception from production and rest. Classification scores on perceived, produced, and rest epochs using an overt-based (dark green) and mimed-based (light green) decoder, Dashed lines indicate the chance level, which is the mean classification accuracy of the distribution generated using random labels

To investigate the differences between brain activity patterns during speech perception and production, we tested if the two brain states could be distinguished from each other and from rest. A separate SVM was trained and tested on all speech perception, speech production, and rest epochs jointly, by classifying each epoch as a perception, production, or rest epoch (not as an individual syllable) using a LoO approach after rest-normalizing every epoch. This was done separately for the overt and mimed produced trials. The three states were highly separable, with average classification accuracies of 91.68% (range 88.31 − 95.30%) and 90.82% (range 86.39 − 95.36%) for overt and mimed speech, respectively (Fig. 4, Supplementary Figure S6). Considering the imbalance in active and rest epochs, the chance level of decoding was well above the theoretical chance level of 33.33% (100 divided by three classes). For both manners of articulation, the ability to classify the three states was above chance for all subjects (*p* <.01, mean chance level 68.34% and 68.39% for overt and mimed speech, respectively).

## Discussion

Recent developments in speech decoding are very promising for individuals who can no longer speak [6–9, 12–14]. However, good results are typically achieved in controlled settings, and not in the daily lives of intended end users. Testing speech BCI reliability in real-life settings is the next hurdle the field needs to take to ensure that BCIs can successfully be adopted by paralyzed individuals. Here, we simulated the scenario where a BCI user is perceiving speech (an intrinsic part of communication) and demonstrate that the perception of speech can be a source of false positive activations of a speech BCI that is trained on produced speech.

The current results confirm that the area frequently used as a signal source for speech decoding BCIs, the ventral sensorimotor cortex, also displays activity during the perception of speech, as was demonstrated before [17–21]. While sensorimotor activation during speech perception is not as widespread as it is during overt and mimed production, the overlap in activity likely contributes to classification of perceived syllables as produced syllables. This was true for decoders based on both overt and mimed speech. Importantly, in line with the speech decoding literature, all electrodes within the grid were used to train the decoding model, not only those that were significantly activated during the production and/or perception of speech. The results show that these false positives arise more during speech perception than during rest periods in the perception task (except when using the mime-decoder after rest normalization). The number of false positive activations differed per participant, but there was no apparent relationship between the number of false positives and the number of electrodes that were significantly activated in both perception and production, nor was there an apparent consistency in the number of false positives relative to the number of electrodes used to train the decoders. Despite considerable overlap between participants in electrode placement, it cannot be excluded that differences in the extent of the brain coverage or the precise positioning of the electrodes affects the occurrence of false positives. In addition, differences in false positives may be related to inherent interpersonal differences, but this remains a question for future research.

Calibration of the brain signals is an often-used practice before conducting a BCI session and aims to normalize the incoming brain signals based on brain signals acquired before a BCI experiment, but this may not always be possible in real-world applications of BCIs. In the current study, calibration using a three-minute rest task recorded independently from the speech tasks did not prevent the occurrence of considerable numbers of false positive classifications due to perceived speech. The argument can be made that these false positives are the result of day-to-day variations in the brain signals, as the speech-production and speech-perception tasks were not necessarily acquired on the same day, which could explain the higher number of false positives during perception-rest epochs compared to production-rest epochs (though this difference was not significant before rest normalization). However, after accounting for the day-to-day variations by normalizing the data on the task-specific rest intervals, the issue of false positive classifications was not resolved. The current results show that this approach to calibration has different effects on the data of different individuals, where for some participants the occurrence of false positives appeared to increase.

To be able to limit the occurrence of false positives caused by speech perception, speech decoders need to be able to distinguish signals corresponding with speech perception from those during production. Though the activity patterns during speech perception resembled those during production enough to be classified as produced speech, there are still differences between the two data. First, activity during speech production was relatively widespread over the SMC, while during perception fewer SMC sites were engaged. Second, perception and production could be distinguished from each other and from rest quite well, as demonstrated by a three-way SVM, with up to 95.36% correct classification. This suggests that the occurrence of false positives during speech perception could potentially be reduced, or even completely removed, if activity patterns during speech perception are considered in the design of speech decoders. One potential way to decrease the occurrence of perception false positives would be to implement a three-step approach, where after the neural detection of speech, the brain activity patterns are classified as being produced or perceived, and only those classified as produced are transferred to the decoder that classifies the actual speech content. The current results suggest that this method could be beneficial, since produced speech could be distinguished from brain activity patterns during speech perception and rest with high reliability in all participants. Another strategy may be to train a decoder that includes speech perception as an additional (9th, in the case of 7 syllables and rest) class, such that neural signal patterns associated with speech perception may be decoded as this perception class. These perception classifications could then be ignored rather than translated in computer output, thereby not eliciting false positives. Both strategies would require the acquisition of additional data for decoder design and training or may need to be updated on a regular basis.

The current study reports on speech perception as a source of false positive activation of a speech BCI, but there are some limitations to this work. First, the numbers of trials on which the decoders were trained are rather small. More trials may be beneficial in decreasing the false positive rates, but this requires further testing. Furthermore, analyses were constrained to high-frequency band signals, which have been shown to reflect local neuronal activity well, but speech decoding may benefit from a combination of frequency bands (see for example [8]). The question remains how the occurrence of false positives is impacted by including signal from different frequency bands. A third limitation is the fact that we classified a limited set of isolated syllables, rather than full words. Longer words benefit from variable word lengths which may make them easier distinguishable on neural data. The current findings may be especially relevant for speech BCIs that allow for spelling based on single phones or phonemes, but further research should be done to assess the occurrence of false positives in word or sentence decoding. Fourth, we classify segments of neural data based on behavioral events (sound or movement onset), rather than using the neural data to detect speech onset. Further research is necessary with speech detection algorithms using neural markers as speech onset to determine whether the occurrence of false positives during speech perception is similar in such situations. Finally, perhaps the most important limitation is that this work is done with individuals who were able to produce speech movements and sounds, whereas speech BCIs are meant to serve people with (severe) communication disabilities. Results obtained with a decoder trained on overt speech might overestimate obtainable decoding performance and/or the occurrence of false positives, as participants were able to hear the result of their speech production. However, also for the decoder trained on mimed speech, which best mimics the situation of intended end-users, syllable decoding performance was above chance and applying this decoder on speech perception data demonstrated a higher occurrence of false positives during perception compared to perception-rest. This indicates that the decoder was not only leveraging brain activity patterns generated by the self-produced auditory feedback. However, it remains important to investigate how these findings translate to paralyzed BCI end users.

The likelihood of a speech BCI being adopted in daily-life depends, among others, on the experienced reliability of the device [15]. The focus of this paper is placed on perception of syllables, but other factors could also induce false positives, such as other movements made by the user, perception of non-speech sounds, contextual information within a sentence [29], or perhaps even imagined or internal speech. To find out which factors may induce false positive activations and thereby decrease reliability of the device, we recommend BCI developers to test their device in real-life settings. If speech BCIs are designed with real-life situations in mind and show high reliability also during two-way conversation, they have a higher likelihood of being embraced by end-users and thus provide added value. Based on our findings, regard for speech perception as a confound during design of speech decoders is likely to enhance reliability of a speech-BCI system.

## Electronic supplementary material

Below is the link to the electronic supplementary material.

Supplementary Material 1

Supplementary Material 2

Supplementary Material 3

Supplementary Material 4

Supplementary Material 5

Supplementary Material 6

## Data Availability

Data is not made publicly available because not all participants consented with public sharing of their anonymous data. Reasonable requests for sharing of the data can be made to the corresponding author.
