# Supplementary material for: Don’t put words in my mouth: speech perception can falsely activate a brain-computer interface
Source: J Neuroeng Rehabil. 2025 Aug 19;22:181. doi: 10.1186/s12984-025-01689-7 (PMC12362870; doi:10.1186/s12984-025-01689-7)
Supplement: Supplementary file 1 — Supplementary Material 1 [file 12984_2025_1689_MOESM1_ESM.pdf]

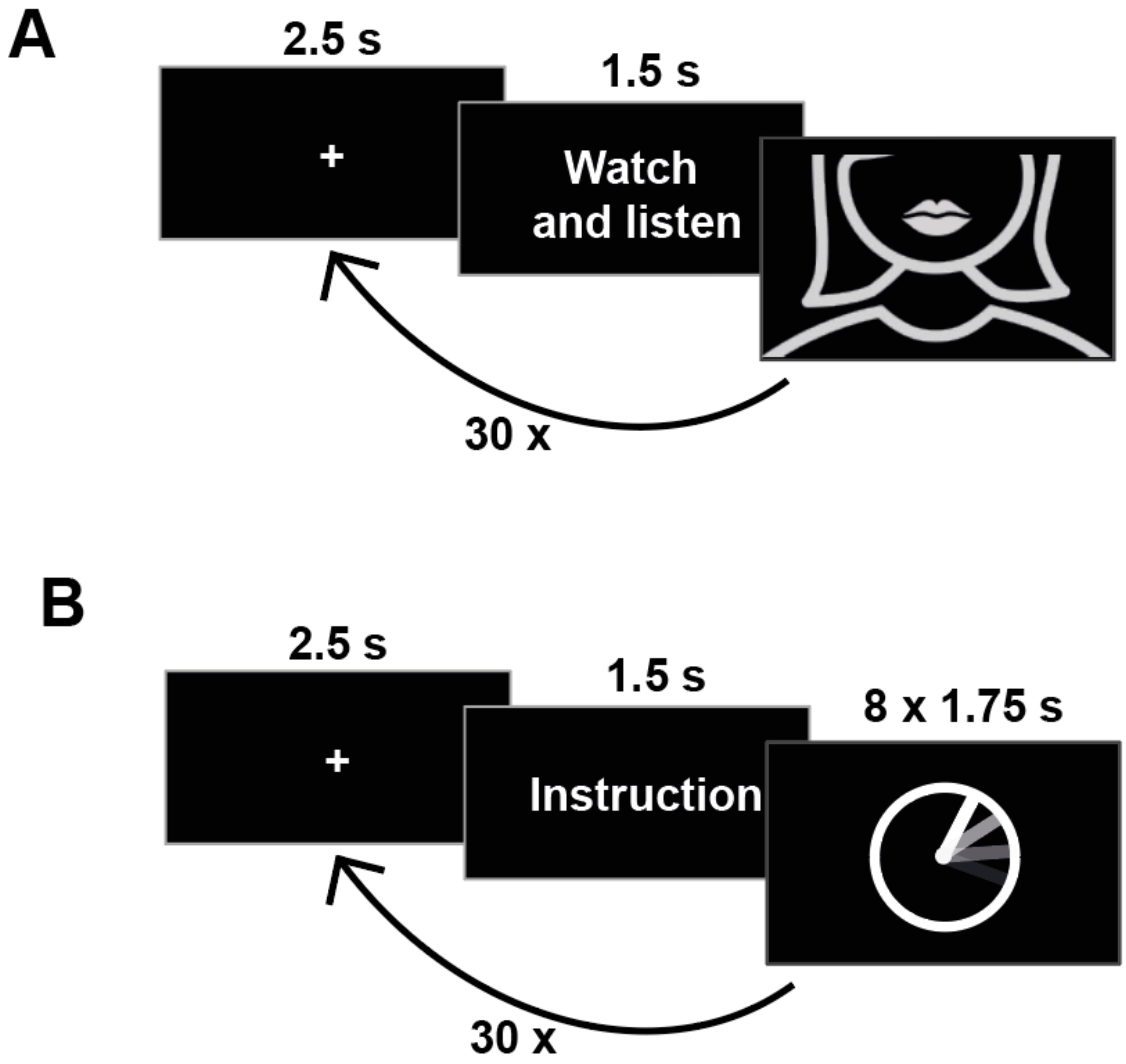

Figure S1: Task design

A) Speech perception task. Rest periods of 2.5 seconds, indicated by a fixation cross were alternated with speech perception. This was one continuous video, in which the lower half of a woman's face was presented while she was producing the syllable sequence. In audiovisual trials, the face was moving and audio could be perceived, whereas in visual-only a still of the woman's face was shown throughout, and in auditory-only conditions no sound was played. Each condition was repeated 10 times, in random order. Prior to each perception trial, the participants were instructed to watch and listen.

B) Speech production task. Participants were instructed to produce the sequence of syllables in either overt, whispered (which was not included in the current study), or mimed fashion, on which they were visually cued. Each condition was repeated 10 times, in random order. A rotating cursor was presented on the screen, and participants were instructed to produce the next syllable in the sequence every time the cursor hit the top of the screen. One cursor rotation was 1.75 seconds.
