# Supplementary material for: Don’t put words in my mouth: speech perception can falsely activate a brain-computer interface
Source: J Neuroeng Rehabil. 2025 Aug 19;22:181. doi: 10.1186/s12984-025-01689-7 (PMC12362870; doi:10.1186/s12984-025-01689-7)
Supplement: Supplementary file 3 — Supplementary Material 3 [file 12984_2025_1689_MOESM3_ESM.pdf]

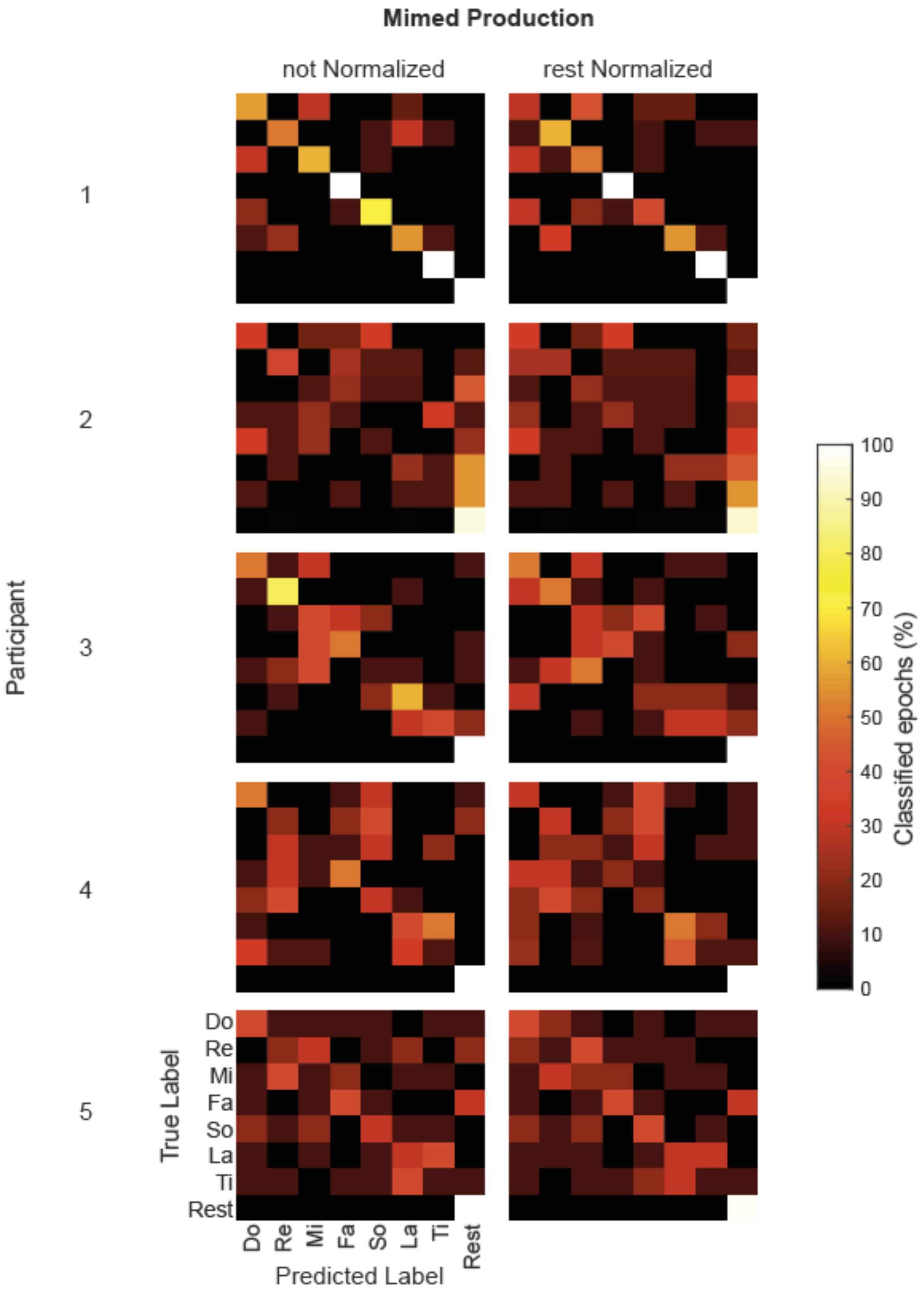

Figure S3 Confusion matrices of classified produced syllables using the decoder trained on mimed production.

For each participant (rows) and for the decoding without and with rest normalization (columns), the confusion matrix of the 8-class classification is shown.
