# Supplementary material for: Don’t put words in my mouth: speech perception can falsely activate a brain-computer interface
Source: J Neuroeng Rehabil. 2025 Aug 19;22:181. doi: 10.1186/s12984-025-01689-7 (PMC12362870; doi:10.1186/s12984-025-01689-7)
Supplement: Supplementary file 4 — Supplementary Material 4 [file 12984_2025_1689_MOESM4_ESM.pdf]

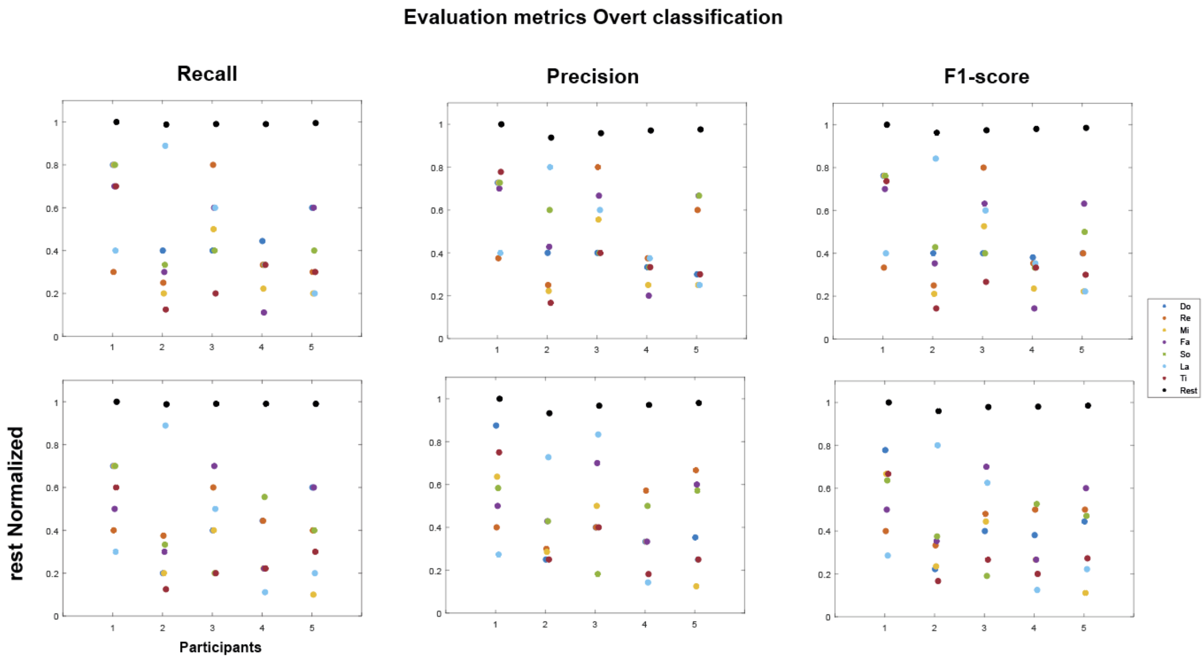

Figure S4: Evaluation metrics for the overt syllable classification

Left, middle, and right columns show the recall, precision, and F1 scores, respectively. The top row shows these metrics for the decoder trained on overt produced syllables prior to rest-normalization, whereas the bottom row shows these metrics after rest-normalizing the data. Participants are represented on the x-axis of each subfigure, and the 8 classes are depicted with separate colors. The score is represented on the y-axis, between 0 and 1.
