# Supplementary material for: Don’t put words in my mouth: speech perception can falsely activate a brain-computer interface
Source: J Neuroeng Rehabil. 2025 Aug 19;22:181. doi: 10.1186/s12984-025-01689-7 (PMC12362870; doi:10.1186/s12984-025-01689-7)
Supplement: Supplementary file 6 — Supplementary Material 6 [file 12984_2025_1689_MOESM6_ESM.pdf]

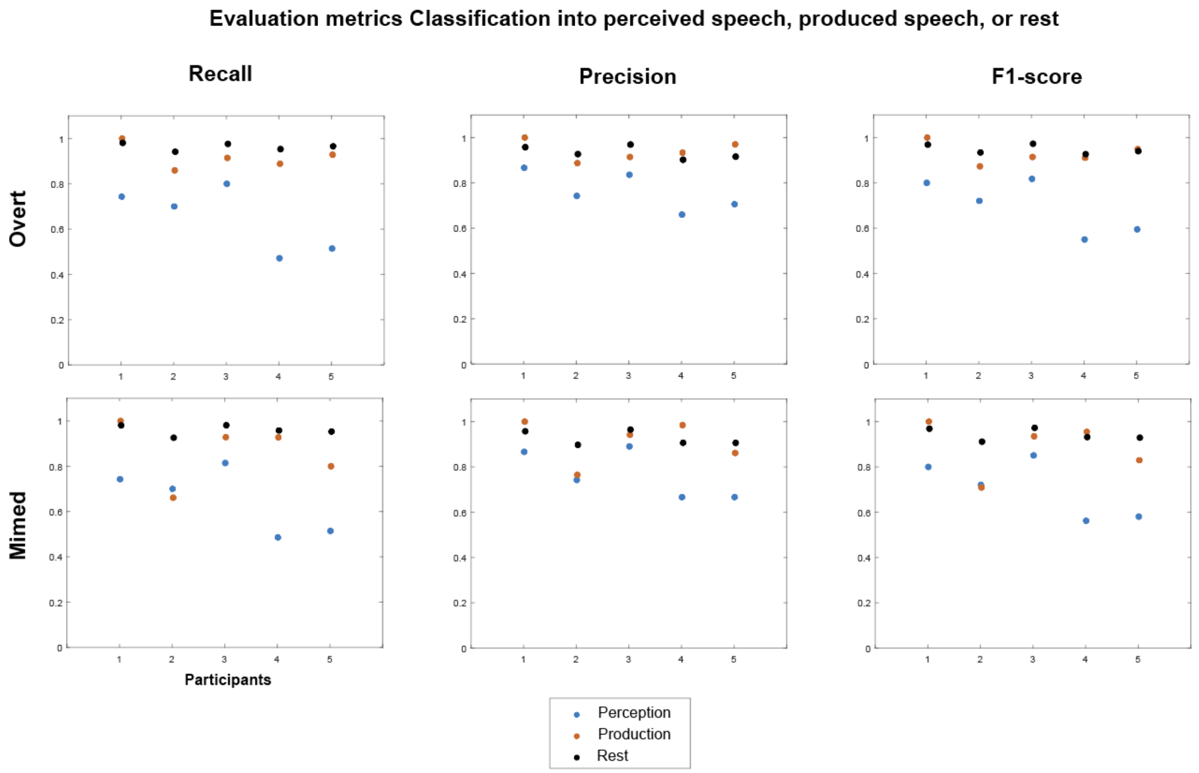

Figure S6: Evaluation metrics for the 3-class classification into perceived speech, produced speech, or rest.

Left, middle, and right columns show the recall, precision, and F1 scores, respectively. The top row shows these metrics for the decoder trained on overt produced syllables whereas the bottom row shows these metrics based on the decoder trained on mimed syllables. Participants are represented on the x-axis of each subfigure, and the 3 classes are depicted with separate colors. The score is represented on the y-axis, between 0 and 1. Note that for a two-step decoder, where first a determination is made whether a brain activity pattern relates to produced speech, perceived speech, or rest, it is important that specifically speech production can be distinguished from the other two classes with high accuracy. Despite lower scores for speech perception in some participants, speech production can be distinguished with high accuracy in all five participants.
